# Deciphering tissue heterogeneity from spatially resolved transcriptomics by the autoencoder-assisted graph convolutional neural network

**DOI:** 10.3389/fgene.2023.1202409

**Published:** 2023-05-25

**Authors:** Xinxing Li, Wendong Huang, Xuan Xu, Hong-Yu Zhang, Qianqian Shi

**Affiliations:** Hubei Key Laboratory of Agricultural Bioinformatics, College of Informatics, Huazhong Agricultural University, Wuhan, China

**Keywords:** spatially resolved transcriptomics, spatial domain identification, spatial information, graph convolutional neural network, autoencoder

## Abstract

Spatially resolved transcriptomics (SRT) provides an unprecedented opportunity to investigate the complex and heterogeneous tissue organization. However, it is challenging for a single model to learn an effective representation within and across spatial contexts. To solve the issue, we develop a novel ensemble model, AE-GCN (**a**uto**e**ncoder-assisted **g**raph **c**onvolutional neural **n**etwork), which combines the autoencoder (AE) and graph convolutional neural network (GCN), to identify accurate and fine-grained spatial domains. AE-GCN transfers the AE-specific representations to the corresponding GCN-specific layers and unifies these two types of deep neural networks for spatial clustering via the clustering-aware contrastive mechanism. In this way, AE-GCN accommodates the strengths of both AE and GCN for learning an effective representation. We validate the effectiveness of AE-GCN on spatial domain identification and data denoising using multiple SRT datasets generated from ST, 10x Visium, and Slide-seqV2 platforms. Particularly, in cancer datasets, AE-GCN identifies disease-related spatial domains, which reveal more heterogeneity than histological annotations, and facilitates the discovery of novel differentially expressed genes of high prognostic relevance. These results demonstrate the capacity of AE-GCN to unveil complex spatial patterns from SRT data.

## Introduction

Spatially resolved transcriptomics (SRT) technologies, such as spatial transcriptomics (ST) (Ståhl et al., 2016), 10x Visium, and Slide-seqV2 (Stickels et al., 2021), can measure the transcript localization and abundance in the dissected tissue area, enabling novel insights into tissue development and tumor heterogeneity (Atta and Fan, 2021; Nasab et al., 2022). Their generated data (i.e., gene expression in tissue locations [spots] and spatial locational information) can be used to decipher the spatially functional regions and cellular architectures in tissues (Maniatis et al., 2021; Marx, 2021; Zeng et al., 2022). However, due to technical limitations (Xu et al., 2022), modeling and integrating the available SRT modalities for accurate spatial domain identification still remain challenging.

Currently, the spatial domain detection methods could be mainly divided into two categories: non-spatial and spatial clustering methods. Some non-spatial methods originally developed for single-cell RNA-sequencing (scRNA-seq) studies, e.g., Seurat (Butler et al., 2018) and Scanpy (Wolf et al., 2018), are also applied in SRT studies. They only utilize the expression profiles to cluster spots while often obtaining domains lacking in spatial continuity to some extent. To address such issues, spatial clustering approaches generally incorporate the additional spatial information into their models. For example, with the spatial prior, BayesSpace (Zhao et al., 2021) and HMRF (Dries et al., 2021) use the Markov random field model (or its variant) to encourage the spatially neighboring spots to belong to the same domain. SpaGCN (Hu et al., 2021) and SEDR (Fu et al., 2021) enable spatial clustering by learning the low-dimensional representation with graph constraints that represent the spatial dependency. STAGATE (Dong and Zhang, 2022) identifies spatial domains by adaptively learning the similarity of neighboring spots via attention mechanisms. Modeling the spatial dependency of gene expression fairly facilitates the discovery of spatial domains with spatial coherence.

Though these methods have provided useful information on the usage of expression profiles and spatial information, they usually depend on single models, which center on either expression data itself or spatially neighboring structure, thus probably resulting in the preferred usage of the focused data type. For example, the non-spatial clustering methods only models the gene expression itself, while the spatial clustering methods often take spatial neighbors prior as a hard constraint to ensure spatial clustering continuity, which may lead to over-smoothing of expression (Huang et al., 2018) and missing subtle spatial regions with a handful of spots. Thus, the rational combination of these different kinds of models can fairly generate more useful representations, enabling better spatial domain detection in SRT studies.

Here, we develop a novel combined model, AE-GCN (**a**uto**e**ncoder-assisted **g**raph **c**onvolutional neural **n**etwork), which combines the autoencoder (AE) and graph convolutional neural network (GCN), for accurate and fine-grained spatial domain identification. Specifically, AE-GCN relies on AE for learning expression data-based representations and GCN for spatial graph-constrained learning. AE-GCN orderly transfers the AE-specific representations to GCN-specific layers and unifies these two types of neural networks for spatial clustering via a clustering-aware contrastive mechanism. In this way, AE-GCN combines the advantages of the two models and takes full integration of the expression data and spatial information during the representation learning process.

We demonstrate the effectiveness of AE-GCN on spatial domain identification and data denoising using SRT datasets generated from ST, 10x Visium, and Slide-seqV2 platforms. In particular, it is validated in two cancer samples that AE-GCN can refine the spatial functional regions and discover novel cancer-associated genes. These results show that AE-GCN is capable of unveiling complex tissue architecture from SRT data.

## Materials and methods

### Overview of AE-GCN

**FIGURE 1:**
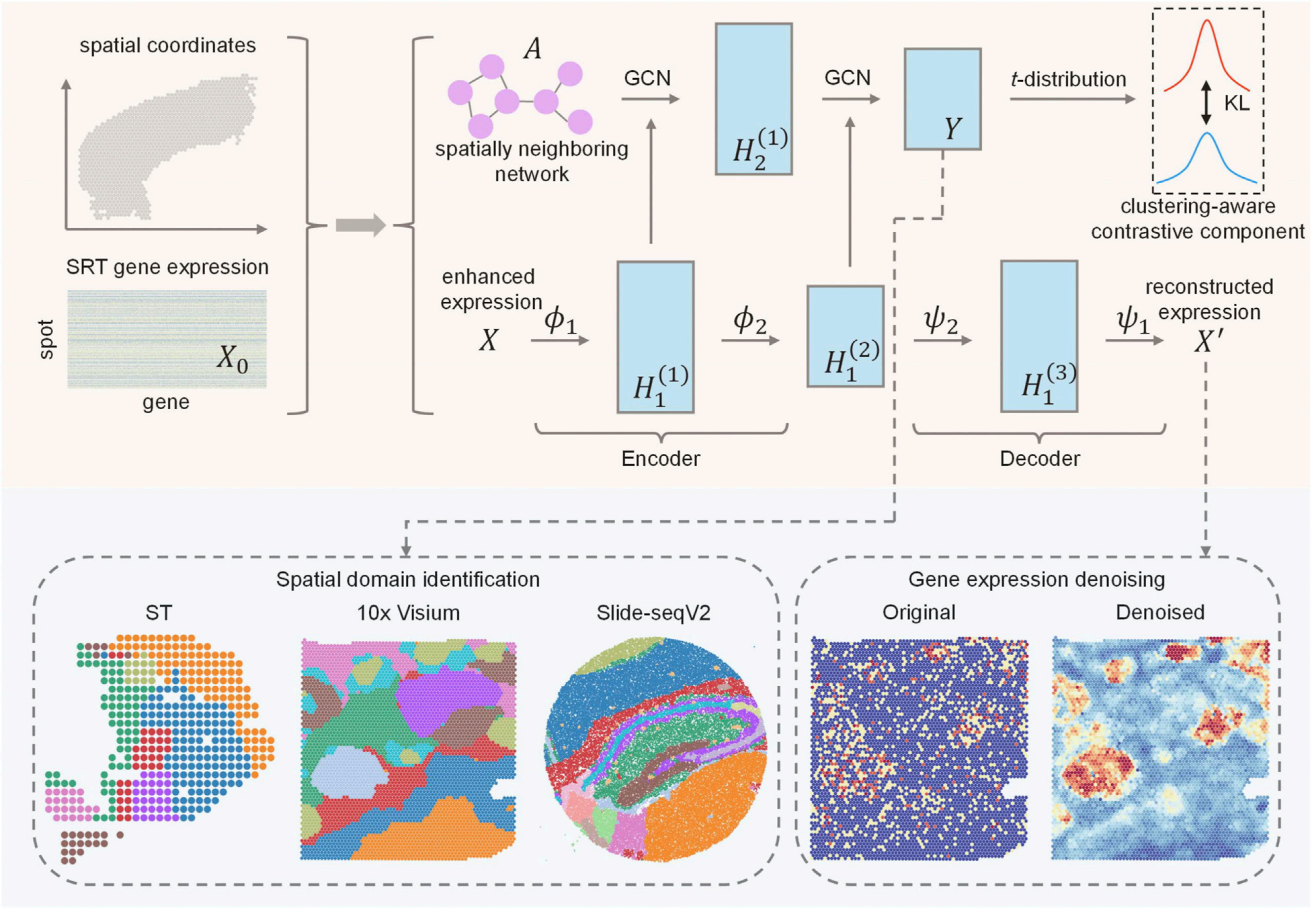
Schematic overview of AE-GCN and its potential applications. Given gene expression and spatial coordinates as input, AE-GCN first builds the spatially neighboring network A and enhances expression X . AE-GCN uses AE to learn representations from the enhanced expression and employs GCN to learn the representations of each layer from the spatially neighboring network A . Then, AE-GCN transfers the AE-specific representations from the encoder to the corresponding GCN-specific layer and learns the combined representation Y . To ensure effective training of the combined deep learning model for clustering, AE-GCN proposes a clustering-aware contrastive module based on the distribution of the representation Y . When AE-GCN reaches convergence, the latent combined representation Y enables AE-GCN to identify spatial domains for different platforms, i.e., ST, 10x Visium, and Slide-seqV2. The reconstructed expression data $X^{\prime }$ serves to denoise expression profiles.

AE-GCN is an integrative scheme that incorporates the AE and GCN learning processes, enabling tasks of spatial domain detection and data denoising (Figure 1).

Given the original expression $X^{0}\in R^{M\times N}$ (where M and N , respectively, denote the number of genes and spots) and spatial coordinates, the spatially neighboring network $A\in R^{N\times N}$ and the enhanced expression data $X\in R^{M\times N}$ are computed as the input of the combined learning process (see Methods). On the enhanced expression data X , AE-GCN employs AE to learn the low-dimensional representation (i.e., AE-specific representation $H_{1}^{\left(l\right)},l=1,\ldots ,L$ , where L is the number of total layers in AE) in each layer. With the spatially neighboring network A , AE-GCN utilizes GCN to learn the graph-constrained representation in each layer (i.e., GCN-specific representation $H_{2}^{\left(l\right)},l=1,\ldots ,B$ , where B is the number of layers in GCN or the encoder of AE). Then, AE-GCN transfers the AE-specific representations from the encoder to the corresponding GCN layer, thus generating the combined representation Y . Additionally, AE-GCN proposes a clustering-aware contrastive module to make the combined representation appropriate for spatial clustering.

When the learning process reaches convergence, the low-dimensional representation (i.e., Y ) of the last layer and the reconstructed expression data (i.e., $X^{\prime }$ ) can be used for downstream analytical tasks. The optimal representation enables AE-GCN to identify spatial domains interoperating with the Leiden method (Traag et al., 2019). The reconstructed expression data serve as the denoised profile, which overcomes the sparsity of the original data to improve differentially expressed gene identification (see Methods).

### Spatially neighboring network construction and expression augmentation

#### Spatially neighboring network construction

Assume that there are original expression matrix $X^{0}$ and spatial locations in the SRT dataset. We first use spatial coordinates and Euclidean distance to calculate the distance between spots and then select the *k*-nearest spatial neighbors of each spot to participate in the subsequent process. In this work, we set *k* = 10 for ST and 10x Visium datasets and *k* = 30 for Slide-seqV2 datasets. Then, we perform principal component analysis (PCA) based on gene expression and select the top p PCs (i.e., $U\in R^{p\times N}$ , default to 15) to calculate the similarity matrix $D\in R^{N\times N}$ between the center spot and its spatial neighbors using cosine metric:

$$\begin{array}{c} D=\exp\left(2-cosine\_dist\left(U\right)\right),D_{ii}=0 \end{array}$$ (1)

Then, the weighted adjacency matrix $A\in R^{N\times N}$ is obtained by normalizing the similarity matrix D :

$$\begin{array}{c} A_{ij}=\frac{D_{ij}}{\sum_{i=0}^{N}D_{ij}} \end{array}$$ (2)

#### Spatial expression augmentation

Limited by the transcript capture rate of SRT technologies, expression data are often sparse and noisy. AE-GCN generates the enhanced expression data X by borrowing the shared information from spatial neighborhood, which can correct low-quality measurements and strengthen local similarity:

$$\begin{array}{c} X=X^{0}+\alpha X^{0}A \end{array}$$ (3)

where the tunable parameter α is flexibly set, and it controls the extent to aggregating expression information from neighboring spots.

### AE component

We employ AE to learn the useful representations from the expression data itself and assume that there are B layers in the encoder and $\left(L-B\right)$ layers in the decoder. Specifically, the learned l th layer representation, $H_{1}^{\left(l\right)}$ , can be obtained as follows:

$$\begin{array}{c} H_{1}^{\left(l\right)}=\phi_{l}\left(W^{\left(l\right)}H_{1}^{\left(l-1\right)}+b^{\left(l\right)}\right),l=1,\ldots ,B \end{array}$$ (4)

$$\begin{array}{c} H_{1}^{\left(l\right)}=\psi_{l}\left(W^{\left(l\right)}H_{1}^{\left(l-1\right)}+b^{\left(l\right)}\right),l=B+1,\ldots ,L \end{array}$$ (5)

where $\phi_{l}$ and $\psi_{l}$ are the activation functions of the l th layer in the respective encoder and decoder. $W^{\left(l\right)}$ and $b^{\left(l\right)}$ are the weight matrix and reconstruction error in the l th layer, respectively. For convenience, we denote the enhanced expression data X as $H_{1}^{\left(0\right)}$ .

The output (i.e., $X^{\prime }=H_{1}^{\left(L\right)}$ ) of the decoder part is obtained through the reconstruction of the input data (i.e., X ) by minimizing the following loss function:

$$\begin{array}{c} L_{res}={\left\|X-X^{\prime }\right\|}_{F}^{2} \end{array}$$ (6)

### GCN component

AE-specific representations, e.g., $H_{1}^{\left(1\right)}$ , $H_{1}^{\left(2\right)}$ , ⋯ , $H_{1}^{\left(L\right)}$ , can denoise data itself and extract valuable information from the data itself, which can effectively reflect expression variation but cannot guarantee the spatial smoothness of the identified domains. GCN can model the spatial structural dependency between spots, which is beneficial to improving the spatial smoothness of the identified domains. Thus, we then transfer AE-specific representations in the encoder into GCN-specific representations and use the GCN module to propagate these AE-specific representations for capturing a more complete and powerful representation. Thus, the GCN-learnable representations can accommodate two different kinds of information: gene expression values and spatial neighborhood structure. The representation learned by the l th layer of GCN, $H_{2}^{\left(l\right)}$ , can be obtained as follows:

$$\begin{array}{c} H_{2}^{\left(l\right)}=\left(1-\mu \right)\phi_{l}\left(W^{\left(l\right)}H_{2}^{\left(l-1\right)}{\tilde{D}}^{-\frac{1}{2}}\tilde{A}{\tilde{D}}^{-\frac{1}{2}}\right)+\mu \;H_{1}^{\left(l\right)}{\tilde{D}}^{-\frac{1}{2}}\tilde{A}{\tilde{D}}^{-\frac{1}{2}} \end{array}$$ (7)

where I denotes the identity diagonal matrix. $\tilde{A}=A+I$ and ${\tilde{D}}_{ii}=\sum_{j}{\tilde{A}}_{ij}$ . ${\tilde{D}}^{-\frac{1}{2}}\tilde{A}{\tilde{D}}^{-\frac{1}{2}}$ is the normalized adjacency matrix. μ is the balance coefficient and is often uniformly set to 0.5. Note that GCN and AE share weights.

Note that we denote the representation (i.e., $H_{2}^{\left(B\right)}$ ) of the last GCN layer as Y . The input of the first layer GCN can be obtained from the enhanced expression data X :

$$\begin{array}{c} H_{2}^{\left(0\right)}=\phi_{l}\left(W^{\left(0\right)}X{\tilde{D}}^{-\frac{1}{2}}\tilde{A}{\tilde{D}}^{-\frac{1}{2}}\right) \end{array}$$ (8)

### Clustering-aware contrastive component

Although we have incorporated the encoder of AE into the neural network architecture of GCN to obtain the combined representation, this representation cannot be directly applied to the clustering problem. Herein, we propose a clustering-aware contrastive module to unify these two different deep learning models for effective spatial clustering.

Specifically, we use student’s *t*-distribution to measure the probability of assigning the spot i to cluster j based on the combined latent representation Y as follows:

$$\begin{array}{c} q_{ij}=\frac{{\left(1+{\left\|y_{i}-\mu_{j}\right\|}^{2}/\rho \right)}^{-\frac{\rho +1}{2}}}{\sum_{j^{\prime }}{\left(1+{\left\|y_{i}-\mu_{j^{\prime }}\right\|}^{2}/\rho \right)}^{-\frac{\rho +1}{2}}} \end{array}$$ (9)

where $\mu_{j}$ is the cluster center by *K*-means on learned representations. $y_{i}$ is the i th column of Y . We regard $Q=\left[q_{ij}\right]$ as the distribution of the assignments of all samples. ρ is the degree of freedom of student’s *t*-distribution.

To optimize the AE-GCN-learnable representation from the high-confidence assignment, we make data representation closer to cluster centers for improving the cluster cohesion. Hence, we calculate the target distribution P as follows:

$$\begin{array}{c} p_{ij}=\frac{{q_{ij}}^{2}/s_{j}}{\sum_{j^{\prime }}{q_{ij^{\prime }}}^{2}/s_{j^{\prime }}} \end{array}$$ (10)

where $s_{j}=\sum_{i}q_{ij}$ is the soft cluster frequency. Each assignment in Q is squared and normalized to produce the target distribution P , which makes the data representation surround the cluster centers closer and helps AE-GCN learn a better representation for the clustering task. By minimizing the KL (Kullback–Leibler) divergence loss between Q and P distributions, the target distribution *P* can help the AE-GCN learn a better representation for the clustering task, i.e., making the data representation surround the cluster centers closer, thus leading to the following loss function:

$$\begin{array}{c} L_{cl}=KL\left(P\left\|Q\right.\right)=\sum_{i}\sum_{j}p_{ij}\log\frac{p_{ij}}{q_{ij}} \end{array}$$ (11)

This design is regarded as a clustering-aware contrastive mechanism, where the P distribution supervises the updating of the distribution Q , and the target distribution P is calculated by the distribution Q in turn. Using this mechanism, AE-GCN can directly concentrate two different objectives: clustering objective and data reconstruction objective, in one loss function. Thus, the overall loss function of AE-GCN is

$$\begin{array}{c} L_{obj}=L_{res}+\beta L_{cl} \end{array}$$ (12)

where β denotes the tunable parameter β>0 and can be flexibly set, which balances data reconstruction and clustering optimization.

### Data collection and general preprocessing

The top 3,000 highly variable genes (HVGs) for 13 10x Visium datasets, one ST dataset, and one Slide-seqV2 dataset are selected using scanpy.pp.highly_variable_genes() from the Scanpy Python package. The log-transformation of the expression profiles is performed using scanpy.pp.log1p() on the original gene expression data.

### Spatial domain detection and gene expression denoising

AE-GCN uses the combined latent representation Y to detect spatial domains by Leiden (Traag et al., 2019) algorithms implemented as scanpy.tl.leiden(). The parameter “resolution” can be adjusted to match the number of the manual annotations.

For the enhanced expression matrix X , AE-GCN aggregates the shared information between each spot and its surrounding neighbors by incorporating prior spatial information into gene expression, which is used to adjust expression values in each spot and enrich spatial local signals. For the reconstructed expression data $X^{\prime }$ , AE-GCN uses AE and GCN to reconstruct the enhanced expression matrix X . By minimizing the reconstruction error, the reconstructed data $X^{\prime }$ can reflect both the spatial local signals and expression measurement global signals. Thus, AE-GCN uses the reconstructed expression data $X^{\prime }$ as the denoised profiles.

### Performance evaluation

We use adjusted Rand index (ARI) (Hubert and Arabie, 1985) and cluster purity (i.e., Eq. 13) (Zhao et al., 2021) to quantify the accuracy of the identified spatial domain and the reference annotations from original publications.

$$\begin{array}{c} cluster\;purity=\frac{1}{N}\underset{c\in C}{\sum }\max_{g\in G}\left|c\cap g\right| \end{array}$$ (13)

where C is denoted as the set of the spatial cluster set and G is regarded as the set of annotated groups. Due to cancer slices with rough annotations (e.g., IDC and PDAC cancer data), cluster purity is specifically used to evaluate the clustering performance on SRT cancer datasets (Zhao et al., 2021).

### Survival analysis

We use bulk expression data with patient survival information to evaluate the prognostic significance of genes via the Kaplan–Meier plotter (Zwyea et al., 2021) in the IDC and PDAC cancer studies.

## Results

### Benchmarking AE-GCN against state-of-the-art methods

We evaluated the ability of AE-GCN to detect spatial domains using 12 human dorsolateral prefrontal cortex (DLPFC) slices generated using 10x Visium. The DLPFC dataset obtained from spatialLIBD (Pardo et al., 2022) is manually annotated as the layered regions by gene markers and cytoarchitecture. The annotations can be considered as the ground truth for benchmarking. Based on this dataset, we compared AE-GCN with the existing state-of-the-art methods, including six spatial clustering methods [i.e., BayesSpace (Zhao et al., 2021), Giotto (Dries et al., 2021), SEDR (Fu et al., 2021), SpaGCN (Hu et al., 2021), stLearn (Pham et al., 2020), and STAGATE (Dong and Zhang, 2022)] and three non-spatial algorithms [i.e., variational autoencoder (VAE) (Kingma and Welling, 2019), Leiden implemented in Scanpy (Wolf et al., 2018), and Louvain implemented in Seurat (Butler et al., 2018)]. The adjusted Rand index (ARI) is used to quantify the similarity between the manual labels and identified clusters, which ranges from 0 for poor consistency to 1 for identical clusters.

**FIGURE 2:**
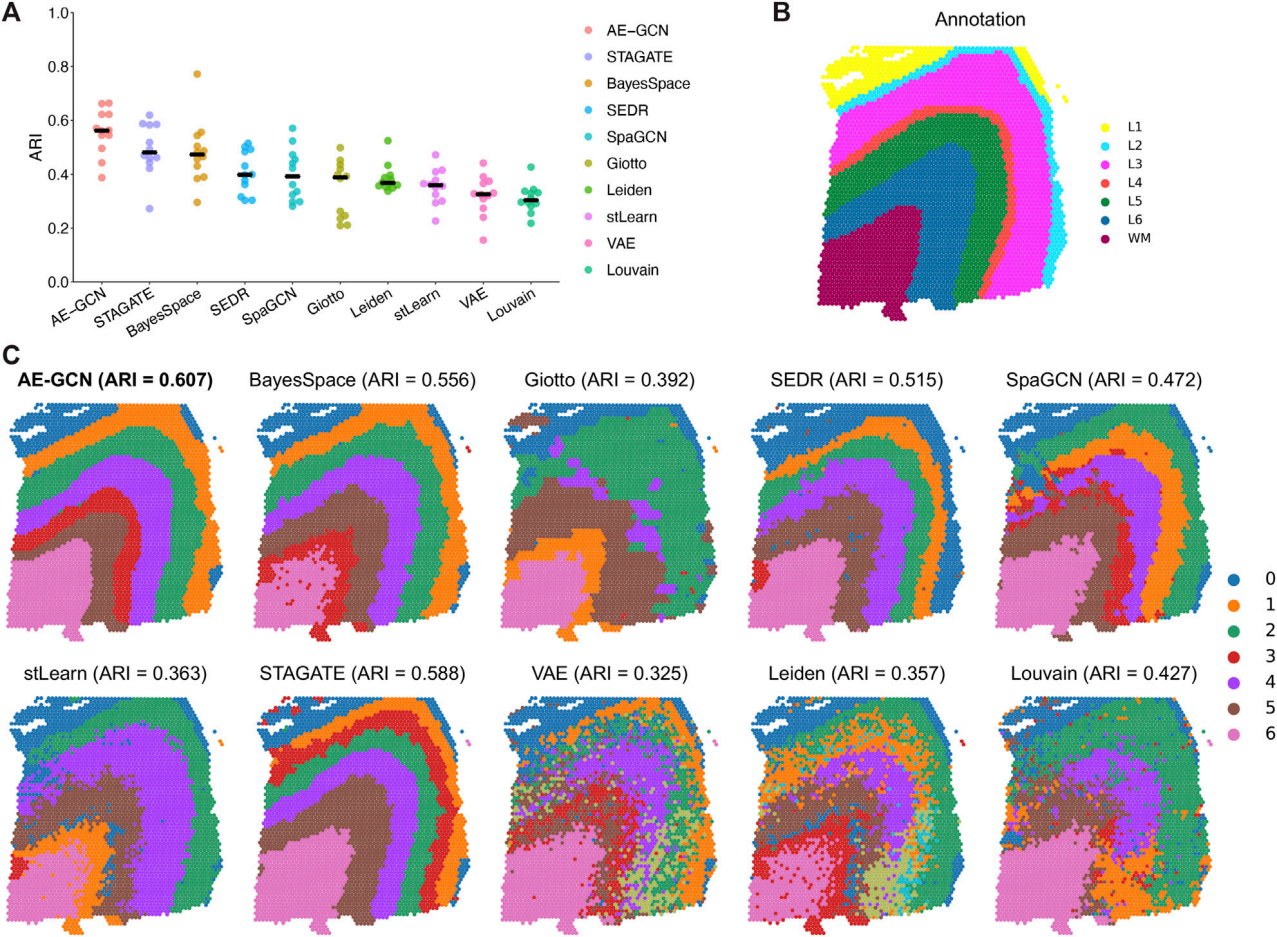
Benchmarking AE-GCN against state-of-the-art spatial domain detection methods. **(A)** Spatial clustering performance is compared using ARI on 12 manually annotated DLPFC datasets from spatialLIBD. The bold line represents the mean ARI value of each approach on all the datasets. **(B)** Slice 151673 with the manual annotation. **(C)** Comparative illustration of the identified spatial domain on slice 151673. The identified spatial domains of each method are distinguished by colors without strict correspondence.

Generally, most of the spatial clustering methods performed better than non-spatial algorithms (Wilcoxon signed-rank test $P<10^{-5}$ , Figure 2A), which showed that the integration of spatial information is necessary to improve the spatial clustering performance. Strikingly, AE-GCN had the highest mean ARI (mean ARI = 0.561) and substantially performed better than the competing methods over the slices (Wilcoxon signed-rank test $P<10^{-8}$ , Figure 2A). Taking slice 151673 as an example (Figure 2B), we found AE-GCN (ARI = 0.623), STAGATE (ARI = 0.588), BayesSpace (ARI = 0.556), and SEDR (ARI = 0.515) delineated the layered regions (Figure 2C). Notably, the partitions from AE-GCN (termed as a deep learning model-combined method) exhibited clearer and less noisy outcomes than those from single model-based methods (e.g., GCN-based SpaGCN and the VAE model).

### AE-GCN reveals fine-grained anatomical regions on mouse hippocampus Slide-seqV2 data

**FIGURE 3:**
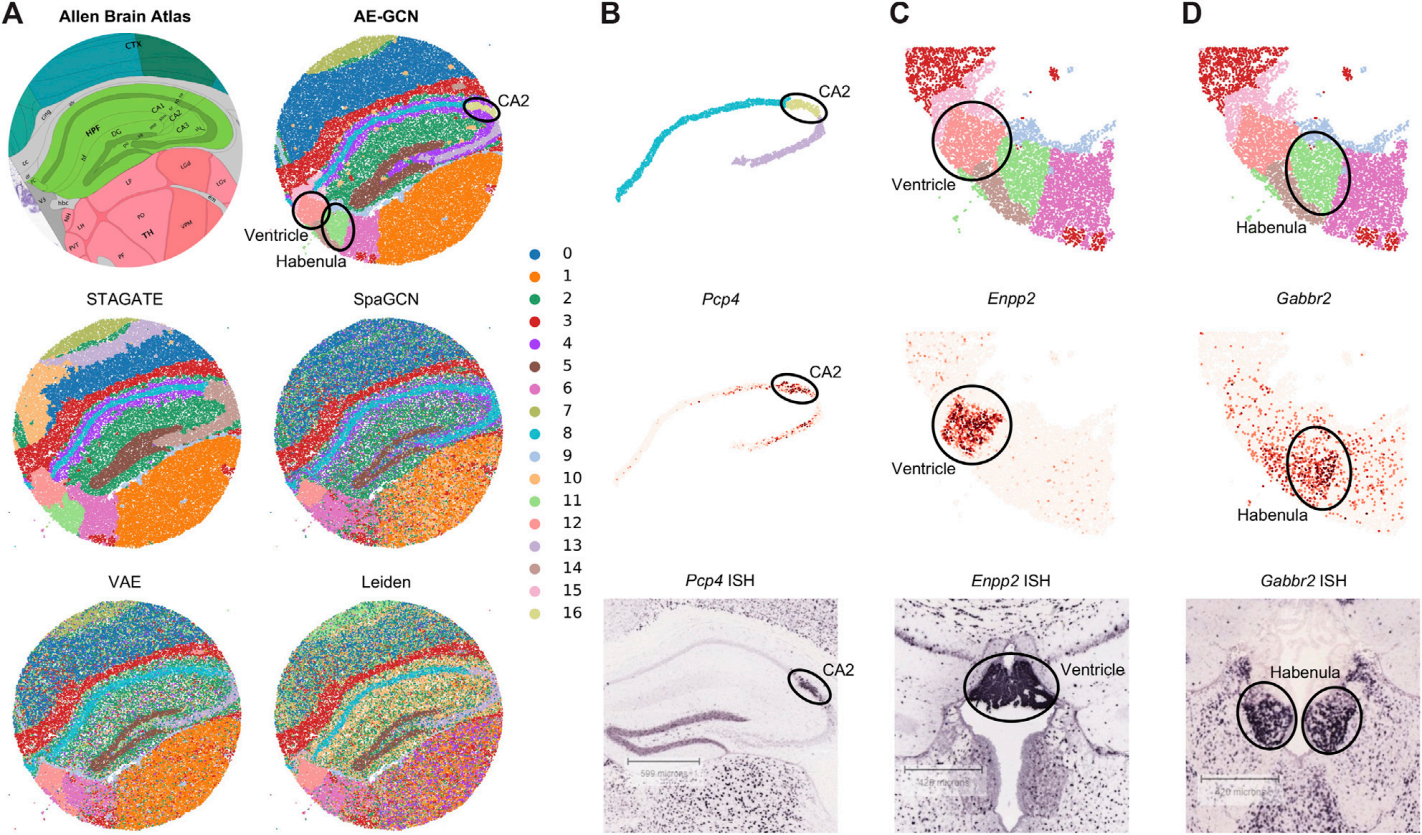
AE-GCN reveals the finer-grained anatomical regions on mouse hippocampus Slide-seqV2 data. **(A)** Corresponding anatomical diagram from the Allen Mouse Brain Atlas and spatial domains identified by each competed method. **(B–D)** CA2 and ventricle and habenula regions (at the top) from AE-GCN partitions are, respectively, validated by the known gene markers (i.e., *Pcp4*, *Enpp2*, and *Gabbr2*) from gene expression (at the middle) and ISH images (at the bottom). The ISH images of *Pcp4*, *Enpp2*, and *Gabbr2* are also obtained from the Allen Mouse Brain Atlas.

To illustrate the effectiveness of AE-GCN on high-resolution SRT platforms, we applied AE-GCN to a mouse hippocampus Slide-seqV2 dataset (*n* = 41,786 spots). Slide-seqV2 can measure gene expression at near-cellular resolution (Stickels et al., 2021) but has lower number of transcripts per location/spot and higher dropouts than the 10x Visium platform. Thus, it poses more challenges for accurately distinguishing tissue structures from the data of high sparsity. To better validate the performance of AE-GCN, we also compared it with other domain detection methods and used the corresponding anatomical diagram from the Allen Mouse Brain Atlas (Sunkin et al., 2012) as the illustrative reference (Figure 3A).

Comparing with the reference, we found that AE-GCN and STAGATE can identify the spatially coherent domains compared to other involved methods. However, AE-GCN performed better to detect the fine-grained structures, such as the cornu ammonis 2 (CA2, AE-GCN domain 16), ventricle (AE-GCN domain 12), and habenula (AE-GCN domain 11) sections (Figure 3A). These sections are delineated with sharper boundaries and higher concordance with the anatomical annotation. We further isolated the focused regions and provided validations from other perspectives (Figures 3B–D). For the CA2 section, which is only detected by AE-GCN, the domain location showed good alignment with the marker gene expression (i.e., *Pcp4* (San Antonio et al., 2014)) and independent *in situ* hybridization (ISH) image (Figure 3B). For ventricle and habenula sections, AE-GCN domains are closer to the shapes of their respective marker expression (*Enpp2* for ventricle (Koike et al., 2006) and *Gabbr2* for habenula (De Beaurepaire, 2018)) or stained regions and match the anatomical shape well (Figures 3C, D). Thus, for higher-resolution SRT data, AE-GCN is capable of effectively unveiling the fine-grained anatomical functional regions.

### AE-GCN accurately discerns tumor regions on human pancreatic ductal adenocarcinoma data

**FIGURE 4:**
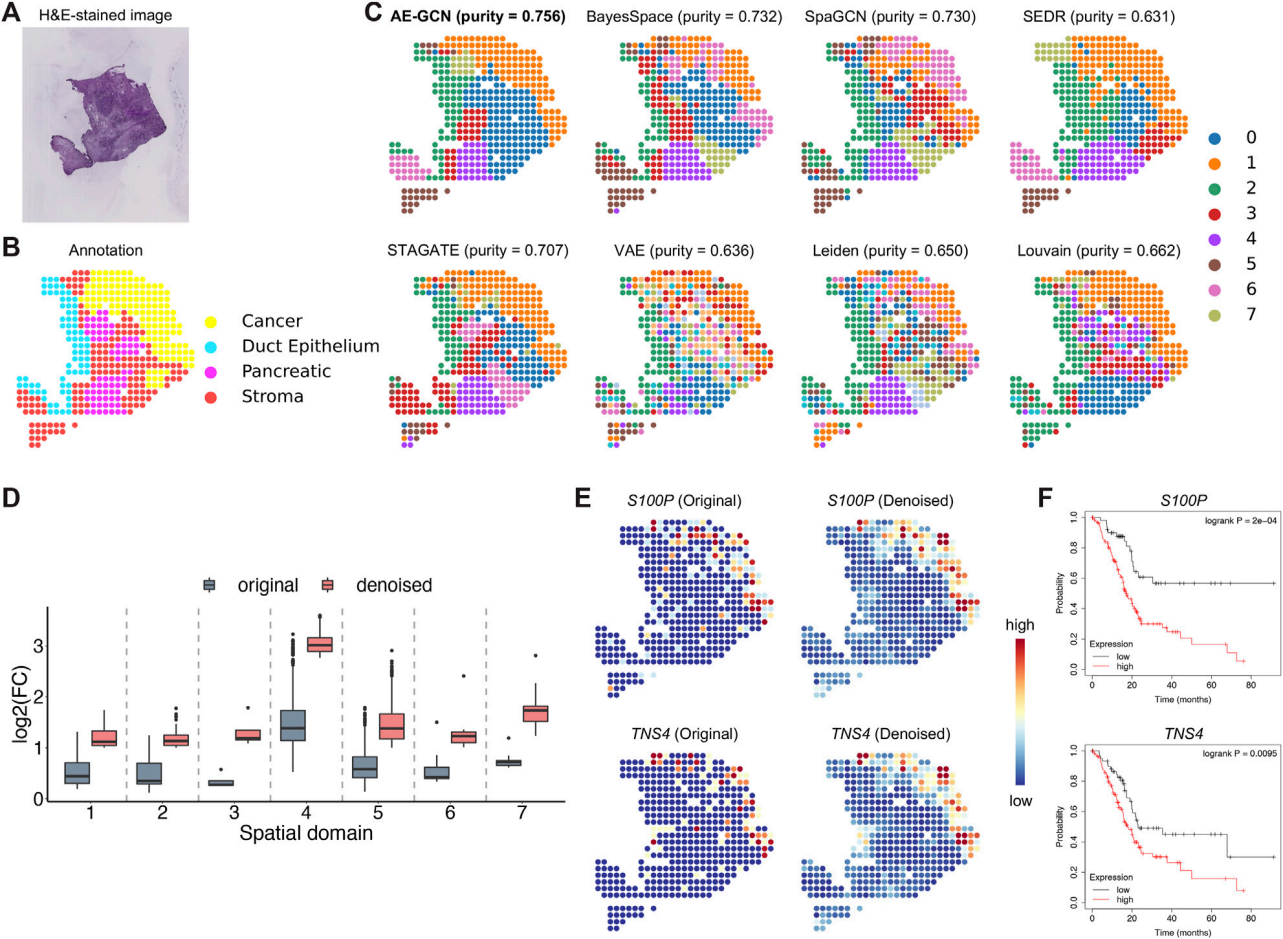
AE-GCN identifies tumor regions on human PDAC ST data. The H&E-stained image **(A)** and the corresponding manual annotation **(B)** are shown as references. **(C)** The identified spatial domains using all the compared methods are distinguished by different colors without strict correspondence. Cluster purity is used to compare the similarity between identified domains and the reference annotation. **(D)** The change in gene differential expression in each domain before and after data denoising. log_2_(FC): the logarithmic value of the gene expression fold change with base 2. **(E)** Spatial expression visualization of selected DEGs (i.e., *S100P* and *TNS4*) before and after data denoising. **(F)** Kaplan–Meier survival curves show the clinical relevance of the identified DEGs (i.e., *S100P* and *TNS4*).

To illustrate the effectiveness of AE-GCN on cancer tissue, we applied AE-GCN to the human pancreatic ductal adenocarcinoma (PDAC) ST dataset (*n* = 428 spots). The histopathological image and annotations were taken as references (Figures 4A, B). We assessed these spatial domain identification methods using cluster purity (see Methods) as the quantitative measure on cancer datasets with rough annotation information. AE-GCN achieved the highest cluster purity (purity = 0.756) and detected more spatially enriched functional regions in tumor tissue than other compared methods (Figure 4C).

Next, we examined whether AE-GCN could provide more insights into the underlying tumor heterogeneity, as data sparsity could hinder other downstream analytical tasks, for example, the identification of differentially expressed genes (DEGs). In this manner, we used the AE-GCN-reconstructed data to denoise the low-quality measurements and evaluated the effectiveness in recovering gene spatial expression patterns. Based on the denoised data, we selected the top 50 DEGs of each domain from the reconstructed data $X^{\prime }$ and compared the log fold change (LFC) of these DEGs before and after denoising (Figure 4D). Overall, the comparison highlights the significant improvement of biological specificity brought by AE-GCN denoising across the identified domains (Wilcoxon signed-rank test $P<10^{-14}$ , Figure 4D). In particular, we found that some DEG expression (e.g., *S100P* and *TNS4*) appeared more spatially smoothed on spots *in situ* (Figure 4E). These two DEGs were validated to be the potential prognostic risk factors for PDAC (Figure 4F). For example, *S100P* is ever reported to be involved in the aggressive properties of cancer cells and associated with poor prognosis (Wang et al., 2012) (Figure 4F); *TNS4* is associated with cancer cell motility and migration, whose high expression can indicate poor prognosis (Sakashita et al., 2008). These results indicate that AE-GCN has the potential to provide the in-depth biological insights into the underlying tumor heterogeneity from the perspectives of spatial domain detection and gene expression pattern recovery.

### AE-GCN reveals more intratumor heterogeneity on invasive ductal carcinoma data

**FIGURE 5:**
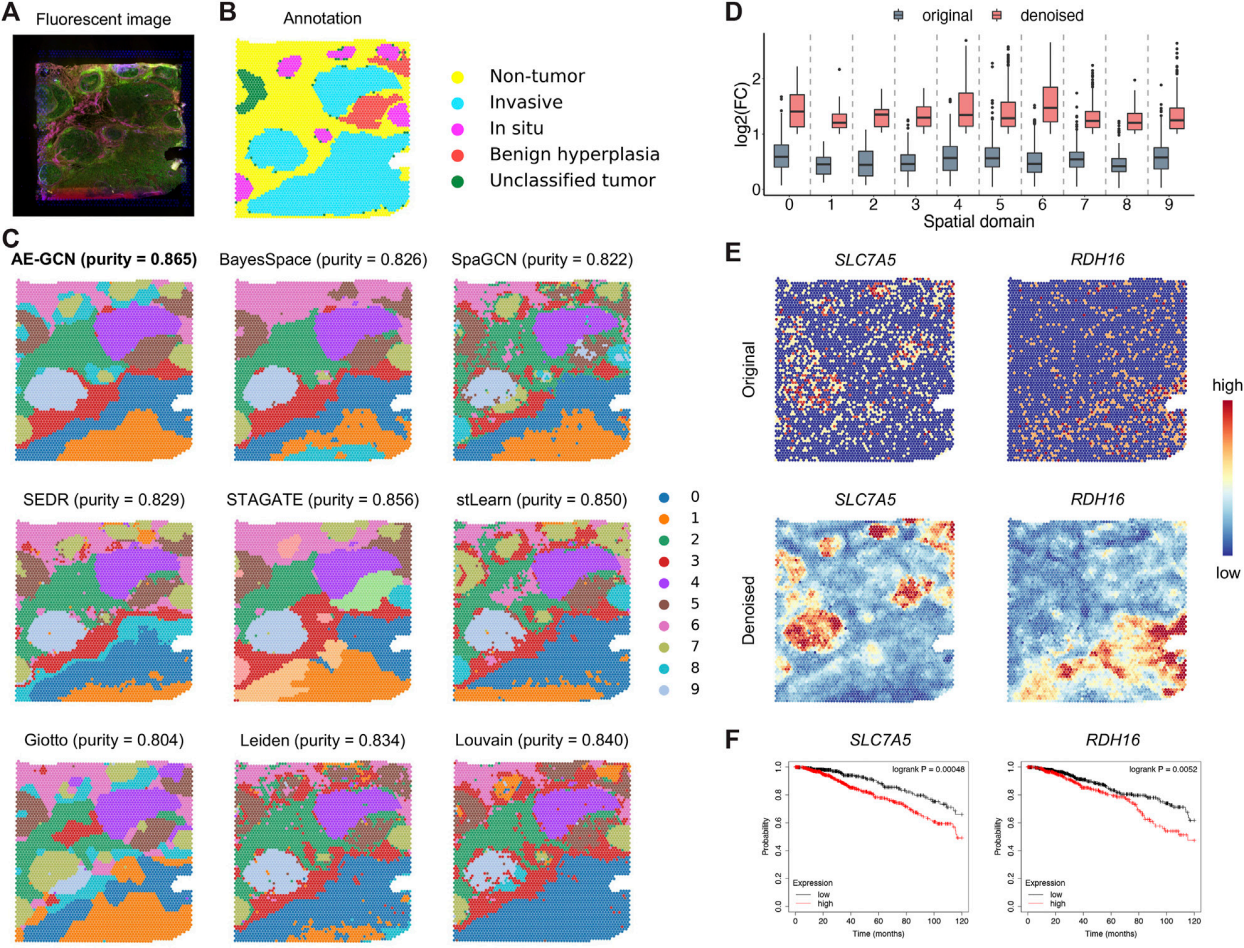
AE-GCN provides more biological insights into intratumor heterogeneity on the IDC 10x Visium dataset. The fluorescent image **(A)** and the corresponding manual annotation **(B)** are shown as references. Each spot is colored due to the annotation label in **(B)**. **(C)** The spatial domains obtained by all involved methods are distinguished using different colors without strict correspondence. Cluster purity is used to compare the similarities between identified outcomes and reference annotation. **(D)** The change gene FC before and after data denoising. **(E)** Spatial expression visualization of the selected domain-specific genes (i.e., *SLC7A5* and *RDH16*) before and after data denoising. **(F)** Kaplan–Meier survival curves show the clinical relevance of the newly identified DEGs (i.e., *SLC7A5* and *RDH16*).

To illustrate the generalization ability of AE-GCN on cancer tissues, we next tested AE-GCN using the invasive ductal carcinoma (IDC) Visium dataset (*n* = 4,727 spots). The histopathological annotations from the original paper (Zhao et al., 2021) were taken as the reference (Figures 5A, B). We found that the identified domains of AE-GCN were highly consistent with the manual annotations (purity = 0.865, Figure 5C). Compared with the domains captured by other methods, the clustering partitions from AE-GCN showed clear spatial separations with few scatter points and high regional continuity.

Then, for functional gene identification, we identified the top 50 DEGs of each cluster from the denoised data $X^{\prime }$ . Similarly, based on the comparison before and after denoising, we found that AE-GCN significantly improves the LFCs of gene expression, revealing more biological specificity across domains, which may suggest the detection of new disease-associated genes (Figure 5D). For example, *SLC7A5* and *RDH16* are two newly found DEGs after denoising, whose spatial expression patterns are greatly enhanced after denoising (Figure 5E). Moreover, the two novel DEGs were shown to be the potential prognostic risk genes for breast cancer via survival analysis of independent clinical data (Figure 5F). Their biological functions in tumors indicate the prognostic relevance from previous studies. For example, *SLC7A5* is reported to involve in tumor cell metabolism and promotes cell proliferation (El Ansari et al., 2018). *RDH16* affects retinol metabolism to participate indirectly in breast cancer occurrence and progression (Gao et al., 2020). The application, along with the PDAC case, demonstrates that AE-GCN can unveil cancer heterogeneity from SRT data, enabling the discovery of novel spatial patterns of both samples and genes.

## Discussion

Spatially resolved transcriptomics technologies measure gene expression on each spot while preserving spatial context, which can support computational methods to identify functional regions of tissue and further resolve organizational heterogeneity. The combined modeling of gene expression and spatial information enables the improved identification accuracy of spatial domains, especially for complex spatial architecture, e.g., tumor microenvironments. In this paper, AE-GCN combines the autoencoder and graph convolutional neural network to achieve effective latent representations from expression data itself and spot neighboring structure. The superiority of AE-GCN is shown not only on the accurate and fine-grained identification of spatial domains for multiple SRT platforms but also on the recovery or identification of gene spatial expression patterns. In particular, the application on cancer slices (i.e., IDC and PDAC) demonstrates that AE-GCN reveals more functional regions and novel cancer prognostic genes for interpreting cancer heterogeneity, suggesting that AE-GCN has great capability of unveiling tissue heterogeneity from SRT data.

The effectively combined modeling is key to the superiority of AE-GCN in the SRT study. Generally, AE models learn the representations from expression data itself, while GCN models learn the structured representations from the sample graph structure by providing an approximate second-order graph regularization, which may suffer from over-smoothing issues. AE-GCN combines the characteristics of these two deep learning methods and integrates them to learn effective representations so that AE is used to weaken the problem of overfitting while simultaneously learning the structured representations in GCN. Additionally, the proposed clustering-aware contrastive module in AE-GCN further promotes the combined model from processes independent of clustering targets to the model that achieves effective spatial clustering. Thus, AE-GCN can not only effectively use the information of the expression data itself but also reasonably regularize the learned information from expression data by spatial structure between spots, which has better advantages than the spatial domain detection methods based on a single-model design in SRT studies.

Currently, AE-GCN only models gene expression and spatial information from SRT data and cannot utilize histological images which are also provided by several SRT technologies, e.g., 10x Visium. Although some methods have used histological images in spatial domain detection, histological images are mainly used to enhance the quality of expression data and lack of modeling image data separately, e.g., stLearn (Pham et al., 2020). Compared with expression data and spatial information, histological image data are one type of modalities more suitable for deep learning modeling. The future work to extend AE-GCN is to integrate deep learning models for each multi-modal data characteristic (i.e., gene expression, histological images, and spatial information) to improve the performance of current methods in SRT research.

## Data Availability

The datasets presented in this study can be found in online repositories. The available web resources include human DLPFC datasets (available in spatialLIBD package), mouse hippocampus Slide-seqV2 dataset (https://singlecell.broadinstitute.org), human IDC 10x Visium dataset (https://www.10xgenomics.com/resources/datasets) and human PDAC ST dataset (https://www.ncbi.nlm.nih.gov/geo/query/acc.cgi?acc=GSE111672). Python source code of AE-GCN is available at https://github.com/zccqq/AE-GCN.
